# Family aggression in a social lizard

**DOI:** 10.1038/s41598-017-03531-0

**Published:** 2017-06-14

**Authors:** Thomas Botterill-James, Ben Halliwell, Simon McKeown, Jacinta Sillince, Tobias Uller, Erik Wapstra, Geoffrey M. While

**Affiliations:** 1 School of Biological Sciences, University of Tasmania, Private Bag 55, Hobart, Tasmania 7005 Australia; 2 Department of Biology, Lund University, Sölvegatan 37, 223 62 Lund, Sweden; 3 Edward Grey Institute, Department of Zoology, University of Oxford, South Parks Rd, OX1 3PS Oxford, United Kingdom

## Abstract

The evolution of family living is underpinned by conflict and cooperation between family members. While family groups can be maintained by reducing conflict between parents and offspring, interactions between siblings may play an equally important role. Here, we compared the level of aggressive interactions between siblings to that between parents and their offspring in the family living skink *Liopholis whitii*. Aggressive interactions occurred much more frequently between siblings and between fathers and offspring than between mothers and their offspring. These results suggest that ecological and social conditions that reduce conflict between siblings and between males and offspring will be fundamental in the evolutionary maintenance and diversification of family living in these lizards.

## Introduction

The evolution of family living is mediated by reduction in within-group conflict. Research in this context has largely focused on parent-offspring conflict and specifically the factors that affect the costs and benefits of prolonged parental investment for both parents and offspring^1, 2^. However, conflict between siblings may be just as important, or more so, than conflict between parents and offspring for mediating family life^3–5^. Indeed, conditions that reduce conflict or facilitate cooperation between siblings can stabilise family life, allowing the emergence of larger family groups^6^. Conversely, conditions that increase conflict between siblings can result in the dissolution of family living^7, 8^. Yet, the relative roles of parent-offspring vs sibling-sibling conflict for the initial origins of family life are not well understood.

Here we compare the levels of parent-offspring and sibling-sibling aggression (as a proxy for conflict) in the social skink *Liopholis whitii*. *Liopholis whitii* live in small family groups characterised by a long-term adult pair bond and prolonged associations between parents and offspring^9, 10^. These prolonged associations involve semi-independent﻿ offspring delaying dispersal and parents tolerating offspring within their core home ranges, sometimes for up to several years^9, 10^. The prolonged association with parents provides several benefits to offspring, including access to resources and protection from infanticidal conspecifics^11^. Importantly, these parent-offspring associations are facultative and there is considerable variation in their strength; from no association between parents and offspring to associations between parents and multiple cohorts of offspring^9, 10^. Furthermore, while there are no costs of associating with offspring for parents^11^, offspring appear to pay a considerable cost of associating with their siblings via increased competition for food and shelter (G. While unpublished data, see also ref. 7). This suggests that 1) aggression between siblings should be high compared to parent-offspring aggression, and 2) that variation in the extent of sibling conflict may explain variation in social complexity in this species. Here we report results from a test of the first of the prediction that aggression between siblings is higher than between parents and offspring.

## Methods

### Collection and housing of the study animals

*Liopholis whitii* is a medium sized (75–100 mm snout-vent length (SVL)) viviparous skink that occurs throughout south-eastern Australia^12, 13^. We captured 103 adult lizards (95 females and 8 males) for three separate experiments in 2014, 2015 and 2016. For each of these experiments, lizards were caught from populations on the east coast of Tasmania, Australia (approximately 42°57′S, 157° 88′E). In all cases, lizards were captured in the field and transported in cool, damp cloth bags back to the University of Tasmania. At the University, lizards were weighed (±1 mg), measured for SVL and total length (±0.5 mm) and sexed via hemipene eversion. Each lizard was uniquely toe-clipped to enable individual identification. No individual lizard was sampled for interaction data more than once (either within or between years). Lizards were then housed individually in plastic terraria (30 × 60 × 40 cm) and kept under a 25 W basking light set to an 8:16 hour cycle with overhead lights set on a 10:14 hour light/dark cycle (to replicate light conditions on the east coast of Tasmania, where the lizards were caught). Each terrarium had a basking rock underneath the basking light, with a wooden shelter at the opposite end. Lizards were provided with water daily and food three times a week (*Tenebrio* larvae and fruit puree mixed with protein powder).

At the end of gestation (mid-Jan), female containers were checked twice daily for the birth of offspring. Upon birth, the date of birth, weight (±1 mg), SVL and total length (±0.5 mm) of offspring were recorded. Offspring were then toe clipped for permanent identification. A coloured ‘bee tag’ (Pender Beekeeping Supplies, Cardiff, Australia) was attached at a point on the dorsal side of offspring using non-toxic glue to allow for instant identification of individuals from video footage (see below). We could not sex the offspring at birth since female offspring retain their hemipenes until sexual maturity and males and females are otherwise morphologically identical. Thus, all data collection on offspring aggression below was collected independent of information on offspring sex.

### Interaction trials: collection of video footage

We filmed mothers and their offspring in their terrariums for a 2-hour period on the day following the completion of the birth of all of a mother’s offspring (determined via abdominal palpation of the female). All interaction trials started at 0900 hrs (following the 1 hr acclimation to the camera being placed above the terrarium and turned on) and filming was conducted using a GoPro Hero4 camera (California, U.S.A). In the 2016 experiment, we also collected video footage of father-offspring interactions for a limited number of families. Father-offspring interaction trials were run the day after the mother and offspring had been filmed together. Specifically, we removed the mother from the home terrarium on the evening prior to filming the father-offspring trials and replaced her with the father (adult males and females were otherwise always housed separately). Filming then began on the following day at 0900 hrs, i.e., 24 hrs after the mother-offspring interaction trial. Overall, each parent was only used once to provide a measure of either mother or father-offspring aggression, and each offspring provided a measure of sibling aggression and was present for both estimates of parent-offspring aggression. The non-independence arising from using the same litter to provide a measure of both sibling and parent-offspring aggression was accounted for statistically (detailed below). After these trials, animals were either released back into their natural population or into semi-natural enclosures as part of on-going projects.

### Scoring interaction data from video footage

For all videos, we scored interaction data from the second hour of footage (following the 1 hr of acclimation). Specifically, we recorded the number of times individuals bit and chased one another as our measures of aggressive behaviour (see Supplementary Videos 1 and 2 for footage of both behaviours). As these two variables were highly correlated (Spearman’s rank correlation = 0.79, p < 0.01), we chose to select one variable for our analysis of differences between parent-offspring and sibling aggression. We decided to analyse the number of chases per hour as our measure of aggression over number of bites per hour. This choice was made for two reasons. First, bites are harder to accurately record than chases. Bites occur quickly as a single motion whereas a chase is composed of a series of small events; specifically, an individual switching from being still or crawling to running directly at a second individual who flees in response; this is distinct from any other type of interaction we have observed in *L. whitii* and therefore it is virtually impossible to record a false positive for chasing. Second, and more importantly, we believe chasing is more representative of aggressive behaviour for this species. For example, while a bite may not illicit any further indication of aggression, for a chase to occur, it is necessary for a given individual to actively switch from one behavior (such as basking or feeding) to instead direct energy into the task of aggressing another individual. Further, chases are typically relentless, with a single chase event ending only once the victim hides underneath a shelter. Regardless, when running analyses with frequency of bites as our conflict measure, our results and interpretations were identical.

Across the three experiments we recorded aggressive interactions between mothers and offspring from 95 one hour videos, between siblings from 89 one hour videos (the sample size for sibling interactions was lower than that for mother-offspring interactions as some mothers gave birth to only one offspring), and between fathers and offspring for 8 one hour videos. Thus, our sample sizes for each component of the study are, n = 95 for mother-offspring interactions, n = 89 for sibling-sibling interactions, and n = 8 for father-offspring interactions. For the father-offspring videos we only used data from the father-offspring interactions, as data on sibling interactions had already been collected as part of the mother-offspring recording. However, there were no differences in the levels of sibling aggression when siblings were with their mother vs. their father (z ratio = 0.67, p = 0.91) suggesting no effects of order or sex of the parent present on the levels of sibling aggression.

### Ethics statement

All experimental protocols were carried out in accordance with the Australian code of practice for the care and use of animals for scientific purposes as approved by the University of Tasmania Animal Ethics Committee (project numbers: A15058, A14380 and A14602).

### Data analysis

Differences in levels of sibling and parent-offspring aggression were modelled using a Generalized Linear Mixed Model (GLMM) fit by maximum likelihood. This was run in R version 3.3.0 (R development core team 2016) using the ‘glmmADMB’ package^14^ with the negative binomial family specified to account for overdispersion. Family interaction (sibling-sibling, mother-offspring, or father-offspring) was entered as a fixed effect, clutch size was entered as a covariate, and experiment year (2014, 2015, or 2016) and litter identity were included as non-nested random effects. Litter identity was included to control for non-independence arising from having a litter provide measurements of both sibling and parent-offspring conflict. Post-hoc comparisons were undertaken using the Tukey’s HSD method for p-value adjustment (implemented through the ‘lsmeans’ package^15^). We did not sample males in the 2014 and 2015 experiments and for the 2016 experiment there were extremely low levels of mixed paternity (only two litters^16^). Therefore, we unfortunately could not conduct any formal tests of the role that relatedness may play in mediating conflict (see discussion).

## Results and Discussion

**Figure 1:**
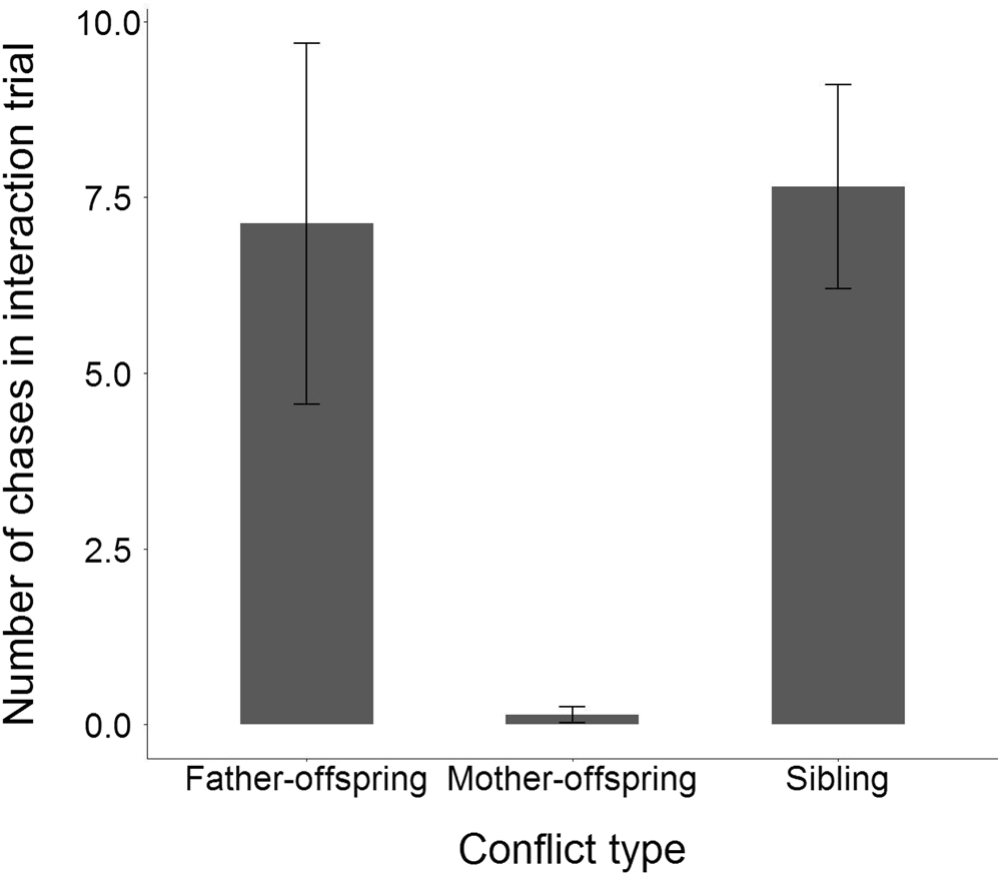
The level of aggressive behaviour, measured as chasing behaviour, between mothers and their offspring, fathers and their offspring and between siblings within *Liopholis whitii* families. Error bars represent one standard error of the mean.

Family dyads differed significantly in the extent of aggressive behaviour (Wald’s χ^2^_(2)_ = 24.33, p < 0.01). Chasing occurred between siblings in 57.3% of interaction trials, fathers chased offspring in 75% of trials whereas mothers only chased offspring in 3.2% of trials. Post hoc analyses revealed that both sibling-sibling and father-offspring aggression were significantly higher than mother-offspring aggression (z ratio = 8.06, p < 0.01 and z ratio = 4.41, p < 0.01 respectively; Fig. 1) but did not differ significantly from each other (z ratio = −0.64, p = 0.79; Fig. 1).

These results suggest that sibling aggression is a key component of *Liopholis whitii* family life. This is consistent with other family living species, particularly birds and mammals, where high levels of aggression between siblings characterises the post birth/hatching environment (reviewed in ref. 17). Importantly, these interactions can play an important role in mediating the composition of the family groups via effects on offspring growth and survival^17^. Unlike many birds and mammals, *Liopholis whitii* offspring are semi-independent at birth, so the most likely consequence of sibling aggression is that it influences the extent to which individuals disperse out of the natal home range as well as the potential identity of dispersers. Such sibling aggression could therefore be a key mechanism determining the considerable variation in family structure observed in *L. whitii* populations^9, 10^. For example, high levels of sibling aggression could explain why only one offspring usually remains within the parental home range despite average litter sizes of 2–3, as observed in some populations of *L. whitii* ^9^. In contrast, low levels of sibling conflict may facilitate the emergence of larger family groups that consist of several offspring remaining within the parental home range or family groups in which multiple cohorts of offspring remain, as has also been observed in a number of *L. whitii* populations^9, 10^, as well as a number of other *Egernia* species^18, 19^.

Sibling aggression is unlikely to be the sole mediator of family dynamics. Indeed, we found that aggressive interactions between fathers and their offspring occurred as frequently as between siblings. Male tolerance of offspring within their natal home range is therefore also likely to be an important mediator of family dynamics within this system. However, the small sample sizes for male-offspring tolerance suggests that this result should be interpreted with caution. In contrast, we found little evidence of mothers exhibiting aggression towards their offspring. Specifically, we found evidence of mothers being aggressive towards their offspring in only 3 of the 95 trials. This may be because *Liopholis whitii* do not exhibit post-hatching provisioning, in contrast to many birds and mammals, and thus the cost of tolerating offspring for females is low^11^. Nevertheless, mothers may still shape sibling conflict because birth of offspring is often spread out across several days (analogous to hatching asynchrony), which influences the outcome of aggressive interactions between siblings^20^. Hatching asynchrony is important in this respect in birds^17^ and deserves further attention in *Egernia* lizards.

These results suggest the maintenance of family living in White’s skink, and perhaps the evolutionary diversity of social organisation in the *Egernia* lizards more broadly, will be dictated by factors that mediate conflicts between siblings and between fathers and offspring. Such conflicts will be influenced primarily by patterns of relatedness between individuals and the costs and benefits of tolerating other individuals^21^. Within-group relatedness will largely be dictated by patterns of female polyandry whereas the costs and benefits of tolerating other individuals will be a function of the environment, in particular resource availability and predation risk. Both of these factors are likely to be important in *L. whitii*. First, we have previously shown considerable levels of polyandry (~30%) within natural populations of *L.whitii* and that this influences the composition of the family group by promoting enhanced dispersal of extra-pair offspring^9^. Second, experimental manipulations of resource availability have been shown to mediate offspring dispersal and the level and nature of parent-offspring associations^11, 22, 23^. While the behavioural mechanisms mediating these facultative responses have not been directly studied, our results here indicate that social interactions with kin are involved. Studies that manipulate levels of female polyandry and examine the consequences for within group conflict could tease apart the causal relationship between polyandry, within group conflict and the evolutionary diversification of social complexity as inferred by recent, large-scale, comparative analyses^1, 6^.

In summary, our results suggest that stable family living in this and other species of lizard rely on reducing conflict between siblings and between fathers and offspring. Since these conflicts depend on both female polyandry and ecological opportunity, studies that manipulate these factors will generate insights into the processes responsible for the maintenance and diversification of family living both within and between lizard species.

## Electronic supplementary material

Supplementary Video 1

Supplementary Video 2
